# Hidden granuloma in plain sight: primary multimucosal tuberculosis (a case report)

**DOI:** 10.11604/pamj.2026.53.79.50724

**Published:** 2026-02-12

**Authors:** Chaimae Bouhamdi, Meryem Soughi, Abir Bouhamdi, Bouchra Amara, Nawal Hammas, Zakia Douhi, Sara Elloudi, Hanane Baybay, Fatima Zahra Mernissi

**Affiliations:** 1 Dermatology Department, Hassan II University Hospital, Fez, Morocco,; 2 Pneumology Department, University Hospital Hassan II, Fez, Morocco,; 3 Anatomical Pathology Department, University Hospital Hassan II, Fez, Morocco

**Keywords:** Primary mucosal tuberculosis, extrapulmonary tuberculosis, sinonasal tuberculosis, granulomatous diseases, case report

## Abstract

Primary mucosal tuberculosis is an exceptionally rare extrapulmonary manifestation, representing a diagnostic challenge due to its marked anatomo-clinical polymorphism and paucity of systemic signs. We report a 62-year-old immunocompetent woman with multifocal mucosal facial plaques showing a fluctuating course. Dermoscopy demonstrated a lupoid pattern. Radiologic evaluation revealed sinonasal extension with osseous lysis. Histopathology confirmed confluent Koester follicles with central caseous necrosis. Systemic evaluation showed no pulmonary or visceral involvement, with only a positive tuberculin skin test supporting prior sensitization. Prompt recognition enabled timely antituberculous therapy, preventing further structural and functional sequelae.

## Introduction

Tuberculosis remains a major cause of morbidity and mortality in endemic countries, increasingly presenting with extrapulmonary forms that complicate early recognition [1,2]. Mucosal disease is exceptional and often paucibacillary, leading to diagnostic delay when chronic lesions mimic inflammatory, infectious, or neoplastic conditions [2,3]. We report a primary multimucosal tuberculosis with destructive sinonasal involvement in an immunocompetent woman, highlighting the need for clinical vigilance, dermoscopic assessment, and timely biopsy in persistent mucosal disease.

## Patient and observation

**Patient information:** a 62-year-old immunocompetent woman from a rural area in a tuberculosis-endemic country, engaged in routine manual cattle tending, presented with a two-year history of multifocal pruritic, infiltrated erythematous facial lesions that appeared simultaneously and followed a relapsing-remitting pattern, alternating between active infiltration and partial spontaneous regression, without preceding trauma or cutaneous breach. She also reported progressive widening of the nasal pyramid with increasing nasal obstruction and frontal headaches. The systemic course remained afebrile but was marked by an involuntary 6kg weight loss.

**Figure 1:**
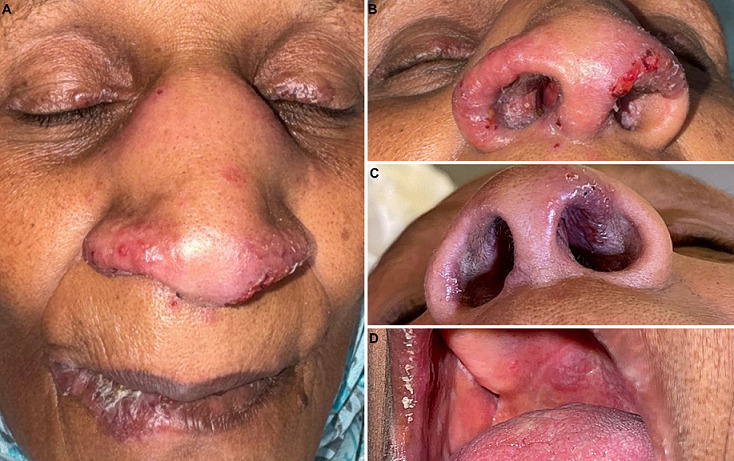
clinical examination showing: A) broadened nasal pyramid with multiple erythemato-violaceous plaques involving the right lower lip, right labial commissure, bilateral nasal mucosa, and medial canthi; B) right nasal cavity showing inflamed mucosa with erythematous nodules and plaques; C) left nasal cavity showing inflamed mucosa with erythematous nodules and plaques; D) hard palate displaying a well-demarcated whitish plaque with erythematous borders

**Figure 2:**
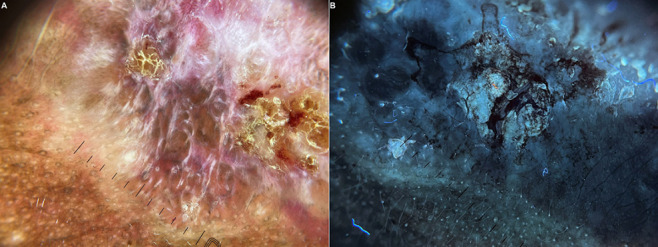
A,B) dermoscopy and ultraviolet dermoscopy showing hemorrhagic and yellowish crusts, whitish lines, white structureless areas, a peripheral whitish halo, and dotted to irregular linear vessels; brown and black structureless areas corresponding to Koester follicles

**Clinical findings:** clinical examination revealed a patient in good general condition, with a broadened nasal pyramid base and multiple firm, oval erythemato-violaceous plaques of variable size, well-demarcated with irregular borders and a non-eroded surface. Lesions involved the right lower lip with mucosal extension, the right labial commissure, the bilateral nasal mucosa, and both medial canthi (Figure 1). No lymphadenopathy was noted. The nasal mucosa was inflamed, displaying intranasal erythematous nodules and plaques, and the hard palate contained a whitish plaque with erythematous margins (Figure 1). Dermoscopy and ultraviolet dermoscopy revealed hemorrhagic and yellowish crusts, whitish scales and lines, white structureless areas, a peripheral whitish halo, and dotted to irregular linear vessels. In both modalities, structures corresponding to histologic Koester follicles were distinctly visible as brown and dark structureless areas, with a positive lupoid pattern. This pattern was appreciable on direct visualization and became more evident with vitropression, supporting a granulomatous process (Figure 2). Based on these findings, mucosal sarcoidosis, mucocutaneous leishmaniasis, peri-orificial cutaneous tuberculosis, and granulomatosis with polyangiitis were considered.

### Diagnostic assessment

Baseline evaluation confirmed an immunocompetent status, with normal hematological parameters and negative HIV serology. A biopsy from the right labial commissure revealed a dense dermal granulomatous infiltrate composed of epithelioid histiocytes and Langhans-type multinucleated giant cells admixed with lymphocytes. The tuberculoid granulomas were confluent and exhibited focal incipient caseous necrosis. Ziehl-Neelsen staining failed to demonstrate acid-fast bacilli.

**Figure 3:**
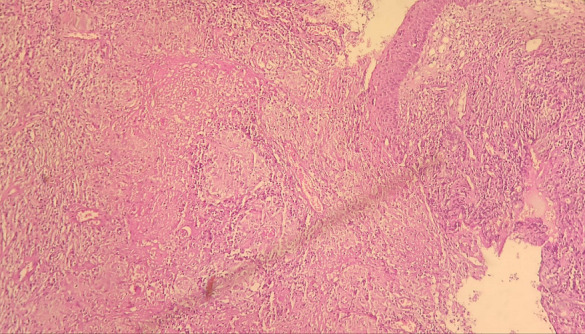
stratified squamous mucosa with a lamina propria showing a granulomatous inflammatory reaction composed of confluent epithelioid and Langhans-type multinucleated giant-cell granulomas with central caseous necrosis (H&E x 100)

Nasofibroscopy disclosed a septal-based soft-tissue mass arising from the nasal septum and extending into both nasal cavities. Computed tomography of the facial bones showed a hypodense infiltrative lesion centered on the nasal bones and piriform apertures, extending to the nasal septum and reaching the right hard palate, with associated osseous lysis, and opacification of the left maxillary sinus with ipsilateral ethmoidal cell involvement. Nasal mucosal biopsy of this septal mass confirmed confluent epithelioid and Langhans-type multinucleated giant-cell granulomas with central caseous necrosis, consistent with caseo-follicular granulomatous tuberculosis (Figure 3).

Systemic work-up showed no clinical evidence of visceral involvement, a normal chest radiograph, two negative sputum examinations for *Mycobacterium tuberculosis* (Mtb), and a positive tuberculin skin test; an interferon-gamma release assay was not performed due to resource constraints.

**Diagnosis:** taken together, the clinical, dermoscopic, radiologic, and histopathologic findings established a diagnosis of primary mucosal tuberculosis. Given the exclusive mucosal involvement, direct mucosal inoculation of Mtb was considered a plausible mechanism of infection.

**Therapeutic interventions:** management adhered to standard antituberculous regimens for extrapulmonary tuberculosis, with an intensive two-month four-drug phase (isoniazid, rifampicin, pyrazinamide, and ethambutol), followed by a four-month continuation phase with isoniazid and rifampicin.

**Follow-up and outcome of interventions:** at three months of antituberculous therapy, nasal obstruction and frontal headaches had resolved, with partial regression of the mucosal plaques. Treatment remains ongoing.

**Patient perspective:** after being informed of the diagnosis, the patient expressed relief at finally understanding the cause of her facial lesions, especially her nasal obstruction and refractory frontal headaches. She acknowledged the need for prolonged antituberculous therapy and agreed to pursue regular follow-up to monitor clinical improvement. She also agreed to implement preventive measures regarding her routine cattle handling.

**Informed consent:** written informed consent for publication of this case and accompanying clinical, dermoscopic, and pathological images was obtained from the patient.

## Discussion

Tuberculosis (TB) remains the second cause of infectious mortality worldwide, right after COVID-19 and surpassing HIV [2]. It is a chronic granulomatous disease caused by *Mycobacterium tuberculosis*, the predominant human pathogen, whereas *Mycobacterium Bovis* accounts for only 1-5% of human TB cases [2]. Extrapulmonary tuberculosis (EPTB) accounts for approximately 20% of the global TB burden according to recent WHO estimates [1,2]. Notably, the proportion of EPTB among all newly diagnosed cases has shown a steady rise in several national surveillance datasets over recent decades. This epidemiological shift underscores the growing importance of recognizing atypical and non-pulmonary presentations in clinical practice [1,2]. Mucosal tuberculosis represents an exceptionally uncommon form of EPTB, with a reported global incidence ranging from 0.05% to 5% of all TB forms [2-5]. It is most often secondary, resulting from contiguous spread, hematogenous dissemination, or autoinoculation from a pulmonary focus, corresponding to endogenous infection [2-5]. Conversely, primary mucosal tuberculosis resulting from direct exogenous inoculation, in the absence of any visceral or pulmonary disease, is exceedingly rare, with only isolated cases documented in the literature to date [2-5].

The pathogenesis of primary mucosal tuberculosis remains incompletely understood. Its rarity is likely attributable to the multiple innate protective mechanisms of mucosal membranes that act as barriers to organisms´ penetration, including mechanical filtration by nasal vibrissae, mucociliary clearance, bactericidal secretions, and structural integrity of the epithelium [2-5]. Chronic irritation or inflammation - such as in poor oral hygiene, cysts, abscesses, leukoplakia, peri-apical granulomas, or periodontitis - weakens these defenses and facilitates bacillary implantation [2-5]. Any epithelial breach, whether traumatic, inflammatory, or iatrogenic (e.g., dental extraction), may allow direct exogenous inoculation of bacilli [2-5]. In our patient, repeated manual contact with livestock was considered a plausible source of inoculation.

Primary mucosal tuberculosis most commonly affects individuals with compromised immunity or living in precarious socioeconomic conditions, particularly children and young adults, and shows a male predominance [2-5]. Systemic symptoms support clinical suspicion, though uncommon. The marked anatomo-clinical polymorphism, combined with the fact that the entity remains poorly recognized, makes the diagnosis a true challenge, leading to diagnostic delay, allowing progressive extension to adjacent structures, risking significant functional and aesthetic sequelae [2-5]. Any mucosal surface can be affected, most frequently the tongue, then the gingiva, hard palate, palatine tonsils, lips, floor of mouth, and major salivary glands, while sinonasal involvement remains exceptional [2-8]; this was the primary site in our patient, explaining the progressive nasal obstruction and bony destruction observed on imaging. The exceptional rarity of sinonasal involvement further contributes to misdiagnosis and delayed therapeutic intervention [6-10]. Lesions are painful or asymptomatic, solitary or multifocal. The most typical presentation is a unique painful chronic ulcer with well-demarcated yet irregular, indurated and undermined borders, refractory to topical therapies, with a base covered by yellowish inflammatory exudates or bluish-gray granules. Other reported morphologies include firm nodules and plaques, macrocheilitis, verrucous proliferation, and poorly defined papillary-granular growth [2-5]. Nasal involvement may present as a unilateral proliferative, infiltrative, or ulcerative lesion, further complicating the differential diagnosis with neoplastic or granulomatous disorders [6-10]. Dermoscopy under diascopic pressure displays a lupoid pattern, with orange-yellow to violaceous hues darkening and becoming brown over time, and candy-granule-like yellow globules, reflecting dermal tuberculoid granulomas that are active structures that gradually involute into fibrotic scarring before reactivating. This dynamic behavior correlates with the episodic infiltration and partial regression reported in our patient.

Mucosal lesions are typically paucibacillary, and Ziehl-Neelsen staining is positive in only 27 to 60% of cases [2-5,7]. This low microbiological yield contributes to the diagnostic challenge of mucosal tuberculosis and underscores the crucial role of clinicopathologic correlation [2-5,7]. The histopathologic hallmark is Koester follicles, which are confluent tuberculoid granulomas; each granuloma consists of a palisading rim of Langhans-type multinucleated giant cells -formed by the fusion of epithelioid histiocytes with eosinophilic cytoplasm and peripheral, horseshoe-shaped nuclei- surrounded by epithelioid cells, macrophages, and an outer lymphocytic mantle, with a central caseous necrosis, an amorphous eosinophilic necrotic material, occasionally appearing cracked, granular, or homogeneous, sometimes with residual nuclear debris [2-5,7]. In some cases, a concentric layered architecture is appreciable, featuring successive zones of lymphocytes, epithelioid cells, giant cells, and central caseous necrosis [2,6-10]. Management generally follows standard multidrug antituberculous therapy used for extrapulmonary disease, with prolonged regimens often required in destructive sinonasal involvement to prevent structural sequelae and relapse [6-10].

## Conclusion

This case illustrates primary mucosal tuberculosis as an unusual form of extrapulmonary disease with simultaneous involvement of multiple mucosal sites and destructive sinonasal disease in an immunocompetent patient, creating a major diagnostic blind spot in endemic settings. The fluctuating granulomatous morphology, paucibacillary profile, and bone-destructive extension highlight the need for heightened clinical vigilance toward persistent, unexplained mucosal lesions and for prompt dermoscopy-guided histopathologic confirmation when microbiology is noncontributory. A multidisciplinary approach is essential to limit diagnostic delay, secure timely antituberculous therapy, and avert irreversible structural sequelae.
